# Editorial

**Published:** 1875-09

**Authors:** 


### Editorial.

### AMALGAM.

We made note in the August number of the REGISTER of some experiments with reference to amalgam made by Dr. Cutler, given in a paper published in the transactions of the New York Odontological Society. They were not of such character, as to impress one, at all familiar with the subject, very favorably.

On page 22, at bottom of transactions, he gives a formula by Dr. Fletcher, as follows: "Platinum one part, gold four parts, tin and silver equal quantities, 21 parts, six grains of filings took up three grains of mercury.

"In twenty-four hours lost nothing in weight and became quite hard, is white and clean looking and ought to be good for bad front teeth."

Dr. C., does not tell us whether it *is* good or not; if it ought to be good for bad front teeth, why should it not be good for those that are not bad?

Again he says in the next paragraph: "Now as to shrinkage, (as given by Dr. Chas. Tomes,) I think it is more theoretical than actual in practice, if properly done."

What is the difference between theoretical and actual shrinkage? Is theoretical shrinkage no shrinkage at all? This we infer for any shrinkage however slight is actual. It is probable the doctor intended imaginary for the word theoretical. "I have to witness the first case of leakage from shrinkage, when the work has been faithfully performed." A very convenient and necessary proviso.

From the fact that about nineteen out of every twenty of all the amalgam fillings that we have examined, and they would number several thousand during the last thirty years, we are led to conclude that there has been very little faithful work with amalgam. And yet one of the great arguments for it is, that it is so easily used, that almost any one can learn in a very short time to fill teeth with it as good as the best.

Well, in view of the facts, we are ready to grant this, for we are constantly witnessing most significant failures, and in large numbers too, from the hands of those who claim to be well skilled in its manipulation. He further remarks, "The sealing over a filling with wax, would be a desirable thing if practicable, which it certainly is not." We are thankful for the last five words, there are a great many things in this world that would be very desirable, were it not for "old impracticability."

In what way would covering an amalgam filling with wax, make it better? would it prevent contraction? would it prevent oxidation of the alloy or any of its components, after the wax was removed? Dr. C., as a chemist, knows it would not do either. But if there is anything that will make amalgam fillings better, we hope it will be used, even if it is beeswax.

On page 23 the doctor gives some experiments on the effect of heat on amalgam, by the blowpipe. These experiments elicit nothing, that is not entirely familiar to every dentist who works in metals. He again remarks, "Free or uncombined mercury remains in the filling, and does not volatilize at all after firmly setting, and no oxide or salt of mercury is found in the mouth, as mercury is not easily acted upon as shown by experiments which I made while in New Orleans, in 1871."

This statement is in marked contrast with the clearly expressed statements of nearly all the authorities on the subject, for instance, Mr. Henry Watts, in his Dictionary of Chemistry vol. 3, page 884, says: "Mercury remains unaltered when agitated for any length of time with oxygen gas, common air, hydrogen, nitrogen, nitrous oxide, nitric oxide, carbonic acid gas or alcohol; but any foreign metals that may become mixed with it, becomes oxidized by agitation in air or oxygen gas, producing a gray pulvurulent mixture of the oxides of the foreign metals and finely divided metallic mercury. On the other hand, by agitation with water, ether of oil of turpentine, or by trituration with sulphur, sulphide or antimony sugar, grease, etc., even in vacuo, mercury is converted into a gray powder, *æthiops per se*, consisting of small globules of the metal." According to Barensprung, some, at least, of the mercury in gray mercurial ointment, is in the state of black oxide, the quantity being greater the older the ointment.

Now we give this simply as evidence from high authority, as to the facility with which mercury oxidizes under nearly all circumstances; and it is in entire agreement with the experience of every one who has worked amalgams, for there is in almost every instance a "*black powder*," produced by the compounding of the metals forming the amalgam. The amount of this material produced in a given time will considerably vary, owing to the metals used, their condition, the manner in which they are brought together, as well as electrical and atmospheric conditions.

We hope to consider this more fully at another time.

On page 24, middle of first paragraph. "Now if there is any shrinkage at all in amalgam fillings it must be owing to the escape of mercury, allowing the fillings or the grains of the amalgam to contract by some cohesive force of the mercury and alloy, a cohesive force drawing them closer together which is somewhat contrary to the effect of crystallization, which I have been led to believe, characterizes this hardening process, though I am not claiming infallibility of opinion."

It is rather difficult to understand the intent of this passage, but he seems to admit here, the escape of mercury, as accounting for any shrinkage, that may take place in amalgam fillings. They nearly all do contract, therefore mercury escapes from them, either as liquid or vapor.

Evidently from this quotation, the doctor has not fully taken in and comprehended the principles involved in the hardening of amalgam, if he did he certainly would not speak of the filings or grains of the amalgam contracting by some cohesive force of the mecury and alloy.

Now filings or grains do not exist in a perfect amalgam,

*crystals do;* the result of the union of mercury with any metal with which it readily unites, is the formation of crystals; crystalization takes place to the extent of the combination.

Now after all the experiments the doctor has given in this paper and the suggestions he has made, we are surprised at the following statement. "Take any mouth or number of mouths, with decayed teeth in every and all stages of decay, and let any first class operator fill all decayed teeth on one side of the mouth with gold, and all on the other side with the best quality of amalgam, say all back of cuspids, the same skill and care being used in both; then turn loose the patient and await results. In my opinion, based upon observation, the side of the mouth filled with amalgam, in ten or twenty years, will be found to be in a better condition than the other." Then of course, gold as a material for filling teeth, is inferior to amalgam, for certainly that which will mantain the better condition is, the better material. If Dr. C., has full faith in the paragraph just quoted, why does he say in the immediately preceeding sentence. " Now in relation to amalgam and its place or rank as a filling, I unhesitatingly place it next to gold for permanent use." And still he says amalgam is more efficient, it will keep the teeth for ten or twenty years in "better condition" than teeth filled with gold, even when done by " first class operators."

Now we regret very much that Dr. Cutler, wrote this article, and for two or three reasons. In the first place he has failed to do himself justice, as every one, who personally knows the doctor will readily recognize; it has probably been written in great haste and so vagueness and faulty composition characterize many of its passages.

No one occupying such a position as Dr. C. does, can afford to send to the world, such a paper as this. And again the influence of the paper is upon the wrong side of a very important practical subject; as an evidence of which, many in the dental profession who have bestowed but little thought and investigation upon the subject, are using this paper as an argument for the indiscriminate, reckless and mischievous use of amalgam.

This, and such productions, gives to professional charlatans and imposters arguments for the use of a doubtful material, one by the use of which they do incalculable mischief. They refer to Dr. Cutler, as high authority, that amalgam is as good if not better than gold for filling teeth. Dr. C. certainly did not see this phase of the subject in all its force, when he wrote this article.

### EDUCATIONAL.

Within a short time, the dental colleges of the country will begin their annual sessions; and the indications are that the classes will be as large if not larger, than heretofore, this at least is the case with some. This is to be expected; the importance and value of a thorough professional education and training is now more fully recognized than ever before. The agitation of the subject in the journals and in the associations, has had a good influence, drawing attention more and more to the subject.

The time is near at hand, when those who enter the profession must, in order to receive full recognition and fellowship, come with that evidence of preparation and ability, which a good corps of special teachers can give.

And in addition to this, the demand for thoroughly competent operators is on the increase, and to such an extent is this the case that it is a matter of surprise, that a far greater number of students do not enter the course of study with higher aims and aspirations than is usual. It is usually the highest object of the dental student to obtain, by some means or other, graduation; while the truth is that the great majority of students have at the time of graduation, progressed far enough, —made attainments sufficient for a foundation upon which to build a good superstructure.

Students instead of asking, In how short a time can I get through? should ask; how much time can I spend profitably in my studies and work, in preparation for the performance of the duties I propose to assume? He who starts out with the full determination to solve the question, will be successful in what he undertakes.

The great majority of dental students should have three terms of college instruction in addition to private pulpilage; and either during this term, or better, independent of it become conversant with the foundation branches of medical science.

There is room, yes urgent demand, for all who may prepare themselves as we have indicated.

Then let every student who may enter our classes this winter, do so with the fixed purpose of making the highest possible attainments, regardless of the time requisite for its accomplishments. With such students, our colleges would take hold of the work before them with warmer zeal and increased energy.

### AMERICAN DENTAL ASSOCIATION.

The annual meeting of this body was held at Niagara Falls Aug. 3d—6th. This was one of the best meetings of this Association held for several years, and in making this statement we refer rather to the spirit that pervaded it, than to the discussions or papers read, though they were both good, and rather above the average of those of former meetings. There was less urging upon the Association anything like personal interests, or individual enterprises, than upon former occasions.

There was a considerable increase of the permanent membership, and very few if any withdrawals. Several of the solid members were absent, which was a cause of regret to all.

This body is from year to year gaining strength, and permanency, and will undoubtedly accomplish, all and even more than its originators and friends could have anticipated

A most excellent selection of officers was made for the coming year, indeed no better could have been made. As a presiding officer for such a body we think Dr A. L. Northrop of New York has no superior, and indeed all the officers chosen are eminently adapted to there respective positions.

We are asked why we do not publish a report of the proceedings of this body in the REGISTER.

Well we do not do it, because the proceedings will be published in full, and better than we could do it, in the volume of transactions to be published by the authority of the Association. And in the next place, two or three other journals are publishing reports of these proceedings quite as well and perhaps better than we could. And again we have on hand an abundance of good original matter, the early publication of which we think will serve the profession a better purpose, than the production of that which has already been, or will be presented to the profession through three or four different channels. We might have made a report on the spot, sent it to the printer and have issued it the next week, as a sample of unparalleled enterprise, but we doubt if that kind of a demonstration would best subserve the interest of the profession just now, and under the circumstances.

### AMERICAN DENTAL CONVENTION.

The annual session of this body was held at Long Branch, Aug. 10—13. There was quite a large assemblage of the profession and a very profitable and enjoyable time was passed From the report of those who were present, it is very apparent that the work accomplished was equal to if not better than upon former meetings of this Convention, reading good papers and excellent discussions was the order of the day. The subject of dental education, had a large share of consideration. It is always a good indication when men take hold of this subject in good earnest. The next meeting will be held in Philadelphia, and in union, we believe, with the American Dental Association.

